# Clinical Phenotypes and Prognosis of Anti-mGluR1 Encephalitis: A Single-Center Case Series and Comprehensive Literature Review

**DOI:** 10.3390/diagnostics16020321

**Published:** 2026-01-19

**Authors:** Rui Ban, Yueyi Yu, Jingli Jiang, Dongchao Shen, Mange Liu, Siyuan Fan, Haitao Ren, Hongzhi Guan

**Affiliations:** 1 Department of Neurology, Peking Union Medical College Hospital (PUMCH), Chinese Academy of Medical Sciences and Peking Union Medical College, Beijing 100730, China; 2 Department of Neurology, Xuanwu Hospital, Capital Medical University, Beijing 100053, China; 3 Department of Neurology, Beijing Renhe Hospital, Beijing 102600, China

**Keywords:** anti-mGluR1 encephalitis, cerebellar ataxia, immunotherapy, paraneoplastic, geographic variation

## Abstract

**Background/Objectives:** Anti-mGluR1 encephalitis is a rare form of autoimmune encephalitis predominantly manifesting as acute/subacute cerebellar ataxia. We describe a newly diagnosed case series from our center and conduct a comprehensive review of reported cases worldwide to compare clinical manifestations, treatment options, and outcomes. **Methods:** We consecutively identified 11 patients at Peking Union Medical College Hospital, and additionally extracted clinical data from 42 previously published cases identified via PubMed and Google Scholar (search updated to 1 August 2025). Demographics, phenotypes, laboratory findings, imaging, treatment, and outcomes were systematically summarized. This pooled review was not prospectively registered, and extracted data from 21 published articles were analyzed alongside our 11 newly diagnosed cases. **Results:** The integrated cohort comprised 53 patients with anti-mGluR1 encephalitis, including 29 males and 24 females, with patients reported from Asia (*n* = 18), North America (*n* = 11), and Europe (*n* = 24). The median age at onset was 50 years (IQR 29.5–58.5; range 3–81), with North American patients presenting later than their Asian and European counterparts (median 60 vs. 48 and 45 years, respectively; all *p* < 0.05). Disease onset was subacute in most cases (58.7%). Comorbid tumors were present in nine patients, most commonly lymphomas. Clinical phenotypes were classified as pure cerebellar syndrome (*n* = 31), cerebellar ataxia with encephalitic features (*n* = 20), and non-cerebellar presentations (*n* = 2). Baseline severity differed across phenotypes (χ^2^ = 35.7, *p* < 0.001). Regional variability in severity was observed but did not reach significance. CSF analyses revealed pleocytosis in 59% (23/39), elevated protein in 31.3% (5/16), and oligoclonal bands in 52.2% (12/23). MRI abnormalities were detected in 34.7% (17/49) of patients, with 21.9% (7/32) developing cerebellar atrophy on follow-up. Therapeutic strategies varied significantly across regions (*p* = 0.041), with Asian cohorts more frequently receiving long-term immunosuppression, European cohorts favoring combined regimens, and North American cases relying predominantly on first-line therapies. Overall, 65.9% (29/44) of patients clinically improved, 13.6% (6/44) relapsed and 20.5% (9/44) remained unaffected. **Conclusions:** Anti-mGluR1 encephalitis presents with significant clinical heterogeneity, ranging from cerebellar-dominant ataxia to neuropsychiatric or non-cerebellar phenotypes, and demonstrates differences in reported age of onset, disease severity, and therapeutic approaches across publication regions. Our findings underscore the importance of early recognition, sustained immunotherapy, and international collaboration to establish standardized, evidence-based management for this rare but disabling disorder.

## 1. Introduction

Glutamate is an excitatory neurotransmitter and functions through ionotropic glutamate receptors (iGluRs) and G protein-coupled metabotropic glutamate receptors (mGluRs). mGluRs are protein complexes composed of dimers and consist of eight subtypes (mGluR1-8), and are classified into three groups based on their amino acid sequences, pharmacological characteristics, and transduction pathways: mGluR I (mGluR1/5), mGluR II (mGluR2/3), and mGluR III (mGluR4/6/7/8) [1,2].

mGluR1 is predominantly expressed in Purkinje cell dendrites and is also present in parallel and climbing fiber inputs [3]. Autoantibodies targeting mGluR1 are responsible for a rare type of autoimmune encephalitis that predominantly manifests as subacute cerebellar ataxia, the presence of clinical symptoms along with the detection of antibodies in the cerebrospinal fluid (CSF) or serum confirms the diagnosis. The initial treatment involves stepwise escalation with immunotherapeutic agents such as high-dose intravenous glucocorticoids, IVIG, and/or PLEX (plasma exchange) as first-line treatment, while second-line or long-term therapeutics include rituximab, cyclophosphamide and other immunosuppressive medications.

Here, we present a newly characterized case series of anti-mGluR1 encephalitis and integrate it with previously reported cases to refine the clinical spectrum and management landscape of this rare disorder. Beyond expanding sample size, our study advances prior work by incorporating systematic phenotypic clustering, longitudinal MRI assessment, and publication-region comparisons of treatment strategies and outcomes.

## 2. Materials and Methods

### 2.1. Patients and Comprehensive Literature Review

This observational cohort included 11 individuals diagnosed with anti-mGluR1 encephalitis at Peking Union Medical College Hospital (PUMCH)’s Neurology Department between 2014 and 2025. Participants were enrolled from the hospital’s Rare Disease Clinical Research Database. Data encompassed demographics, clinical presentations, imaging findings, laboratory results, treatment approaches, and outcomes. Ethical approval was obtained from the institutional review board, and the written informed consent has been obtained from the patients to publish this paper. The study was approved by the ethics committee of Peking Union Medical College Hospital (JS-891/5 January 2025). A comprehensive literature review was performed by querying PubMed and Google Scholar with the terms “mGluR1,” “autoimmunity,” and “encephalitis,” identifying 21 peer-reviewed articles published from 2000 to 1 August 2025 [3,4,5,6,7,8,9,10,11,12,13,14,15,16,17,18,19,20,21,22,23] (See Supplementary Figure S1). Only English-language publications were included in the literature review. We included published case reports and case series with confirmed anti-mGluR1 antibody positivity and extractable individual-level clinical data. Narrative reviews or publications without sufficient case-level information were excluded. These sources reported on 42 cases of anti-mGluR1 encephalitis. In this study, the term “geographic distribution” was defined as the region of publication of case reports, rather than the patients’ birthplace or residence. This definition follows conventions in bibliometric research, where geographic distribution reflects the origin of publications (e.g., author affiliations or publishing countries) rather than the geographic background of study subjects [24]. The review was not prospectively registered.

### 2.2. Antibody Testing

A tissue-based assay using rat cerebellar sections was applied for screening all suspected cases in our center, followed by confirmation with a cell-based assay (CBA) and a previously validated autoimmune cerebellar ataxia antibody panel. In the 11 patients reported in this cohort, immunofluorescence demonstrated characteristic granular staining within the molecular and granular layers and the cytoplasm of Purkinje cells, as visualized by fluorescence microscopy [25]. Subsequent testing with a comprehensive onconeural and autoimmune cerebellar ataxia antibody panel [Tr(DNER), ITPR1, mGluR1, Homer-3, NCDN, Septin complex, Sez6L2, GluRδ2, AP3B2; EUROIMMUN, Lübeck, Germany] confirmed the presence of anti-mGluR1 antibodies in all 11 patients.

### 2.3. Treatments

Immunotherapy was stratified into first-line, second-line, and maintenance therapies. First-line treatment options included corticosteroids, intravenous immunoglobulin (IVIG), and plasmapheresis, administered singly or in combination. Second-line therapies consisted of rituximab (RTX) and cyclophosphamide (CTX), used individually or together. For long-term management, patients received mycophenolate mofetil (MMF), azathioprine (AZA), tacrolimus (TAC), or hydroxychloroquine (HCQ) [26,27].

### 2.4. Data Analysis

All statistical analyses were performed using SPSS software (version 20.0; IBM Corp., Armonk, NY, USA). Categorical variables were summarized as counts and percentages, and group differences were assessed using Pearson’s chi-square test or Fisher’s exact test when expected cell counts were <5. Continuous variables were tested for normality with the Kolmogorov–Smirnov test. Normally distributed data were expressed as mean ± standard deviation (SD), whereas non-normally distributed data were summarized as median and interquartile range (IQR). Between-group comparisons were conducted using the Mann–Whitney U test for two groups or the Kruskal–Wallis test for three or more groups.

Given the relatively small sample size and sparse data in some contingency tables, Fisher’s exact test was prioritized to ensure statistical robustness. A two-tailed *p* value < 0.05 was considered statistically significant. No formal correction for multiple comparisons was performed because of the small subgroup sizes and exploratory nature of the analysis. Therefore, *p*-values should be interpreted as hypothesis-generating rather than confirmatory.

## 3. Results

### 3.1. Case Series from PUMCH (n = 11)

In this single-center case series of 11 Chinese patients with anti-mGluR1 encephalitis, the median age at onset was 46 years (IQR 37–57, range 23–61), with a slight male predominance (7/11, 63.6%). Most patients (10/11, 90.9%) presented with acute to subacute cerebellar ataxia, and 7/11 (63.6%) exhibited severe disability requiring walking aids or wheelchairs. Non-cerebellar features were infrequent, including dysgeusia in 2 patients (18.2%) and transient fear sensations in 1 patient (9.1%). The duration from symptom onset to diagnosis ranged from 10 days to 1 year (see Table 1 for baseline characteristics).

Brain MRI revealed cerebellar signal abnormalities in 3 patients, while CSF analyses demonstrated pleocytosis in 7/11 (63.6%) but without a consistent pattern of protein elevation or OCB positivity. All patients tested positive for anti-mGluR1 antibodies confirmed by CBA. First-line immunotherapy with high-dose corticosteroids and/or intravenous immunoglobulin was administered in all cases, and 6/11 (54.5%) required escalation to second-line or long-term immunosuppressants such as mycophenolate mofetil or rituximab. Clinical improvement was achieved in all patients (11/11, 100%), although residual deficits—mainly persistent gait imbalance—remained in 7/11 (63.6%).

### 3.2. Pooled Analysis of Institutional and Published Cases (n = 53)

As mentioned above, we collected 42 cases of anti-mGluR1 encephalitis previously reported worldwide [4,5,6,7,8,9,10,11,12,13,14,15,16,17,18,19,20,21,22,23] (see Supplemental Table S1). By combining these published cases with our 11 newly identified patients from PUMCH, we performed an integrated analysis and derived the following conclusions.

#### 3.2.1. Patient Demographics and Clinical Characteristics

**Figure 1:**
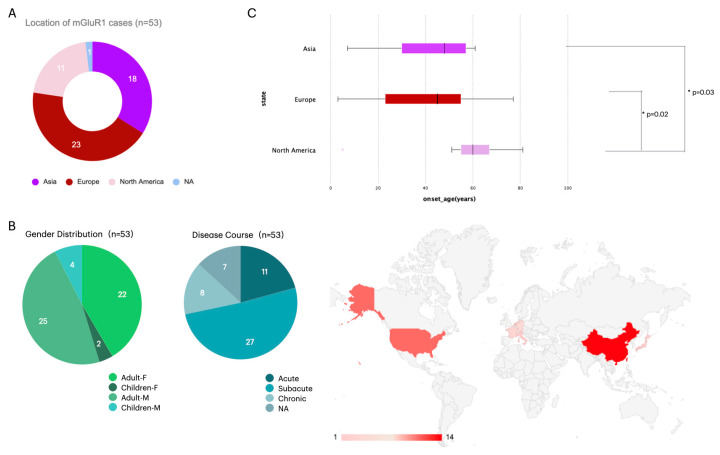
Geographic Distribution and Clinical Characteristics of Patients. (**A**) Geographic distribution of reported cases, including 18 from Asia (China = 14, Japan = 2, Singapore = 1, United Arab Emirates= 1), 24 from Europe (Germany = 4, France = 3, Netherlands = 2, Italy = 1, Austria = 1, others = 13), and 11 from North America (all from the United States). (**B**) Gender distribution demonstrated a slight male predominance, with 29 males (25 adults, 4 children) and 24 females (22 adults, 2 children). Disease course was acute in 11 patients (20.8%), subacute in 27 (50.9%), and chronic in 8 (15.1%), while 7 cases lacked detailed temporal data. (**C**) Comparison of onset age across regions showed a median of 50 years (range 3–81 years), with significantly later onset in North American patients (median 60 years) compared with Asian (median 48 years) and European (median 45 years) cohorts (*p* = 0.02 and *p* = 0.03, respectively). Note: “Geographic distribution” refers to the publication region, not the patients’ origin.

A total of 53 patients with anti-mGluR1 encephalitis were analyzed, including 29 males and 24 females. The geographic distribution, age at onset, and onset patterns of these patients are summarized in Figure 1. Geographic distribution included 18 patients reported from Asia (China = 14, Japan = 2, Singapore = 1, United Arab Emirates = 1), 11 from North America (all from the USA), and 24 from Europe (Germany = 4, France = 3, Netherlands = 2, Italy = 1, Austria = 1, others = 13). The overall median age at onset was 50 years (IQR 29.5–58.5; range 3–81). Disease onset was acute in 11/46 (23.9%), subacute in 27/46 (58.7%), and chronic in 8/46 (17.4%). Notably, cases reported in North American presented at a later age (median 60 years, IQR 54–67) compared with Asian (median 48 years, IQR 28.3–57.0) and European patients (median 45 years, IQR 22.5–55.5) (all *p* < 0.05), suggesting regional variation in age of onset.

Nine patients had a prior history of malignancy, including lymphoma (*n* = 5), seminoma (*n* = 1), leukemia (*n* = 1), sarcoma (*n* = 1), and a dual history of lymphoma and prostate adenocarcinoma (*n* = 1). Most tumor-associated cases originated from Europe (*n* = 7), with two reported from the USA. The latency between lymphoma and neurological onset ranged from simultaneous presentation to 9 years. In contrast, one patient developed seminoma 7 years after encephalitis onset, and another developed prostate adenocarcinoma 2 years after diagnosis of encephalitis, indicating both paraneoplastic and post-neoplastic associations.

#### 3.2.2. Clinical Presentation

Prodromal symptoms were reported in a minority of patients, most commonly fever (15.1%, 8/53), followed by headache (9.4%, 5/53) and fatigue (3.8%, 2/53). The predominant clinical feature was truncal ataxia, observed in 92.5% (49/53), often accompanied by limb ataxia (29/49, 59.2%). Neuropsychiatric and cognitive features were most frequent in European patients (26.4%, 14/53), with lower frequencies in Asia (7.5%, 4/53) and the USA (5.7%, 3/53).

Distinct regional patterns of additional symptoms were observed. Dysphagia occurred in 38.9% of patients reported from Asia and 45.8% of patients reported from Europe but was absent in the North American cohort (*p* = 0.010). Hand or head tremor was more common in Asian patients (38.9%) compared with European (12.5%) and US patients (9.1%) (*p* = 0.031). Dysgeusia was enriched in US patients (27.3%, 3/11) compared with Asian (5.0%, 1/20) and European patients (0/24, *p* < 0.05). Furthermore, apathy and memory loss were more frequently observed in European patients (both 25.0%, 6/24) compared with Asia (0/20, *p* = 0.035). Importantly, no significant differences in disease duration or age at onset were detected between patients with and without these neuropsychiatric features, indicating that geographic variation rather than demographic factors may account for these patterns.

#### 3.2.3. Clinical Phenotypes

**Figure 2:**
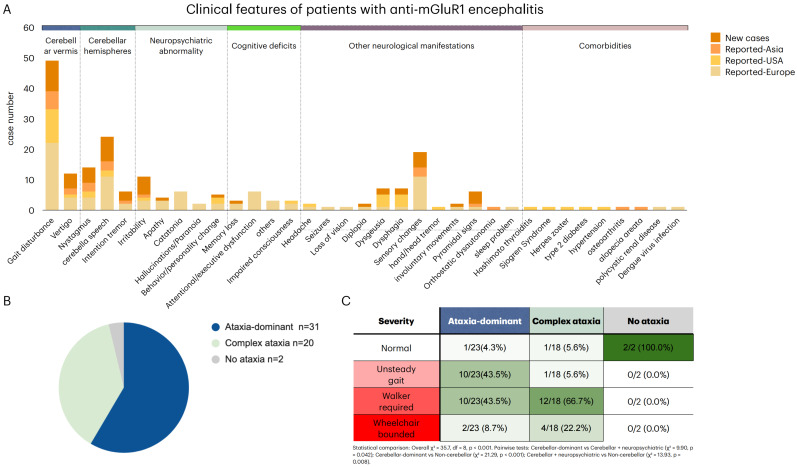
Clinical Manifestations and Phenotypic Classification of mGluR1 Antibody Encephalitis Patients. (**A**) Clinical spectrum of 53 patients, illustrating predominant cerebellar involvement. Gait disturbance and truncal ataxia were the most frequent features (49/53, 92.5%), followed by vertigo, nystagmus, dysarthria, and intention tremor. Neuropsychiatric symptoms—including apathy, behavioral or personality change, and memory loss—were mainly reported in European cases (26.4%, 14/53), whereas dysgeusia was more common in North American patients (27.3%, 3/11). (**B**) Based on symptom constellations, patients were classified into three major clinical phenotypes: (1) Ataxia-dominant phenotype (*n* = 31)—predominantly cerebellar signs including gait ataxia, imbalance and titubation. (2) Complex ataxia with encephalitic features (*n* = 20)—cerebellar syndrome accompanied by cognitive decline, language impairment, behavioral/psychiatric symptoms or altered mental status. (3) Non-cerebellar phenotype (*n* = 2)—presentations lacking dominant ataxia, including seizure-associated or atypical neurological manifestations. (**C**) Severity of ataxia was stratified as normal, unsteady gait, walker required, or wheelchair-bound, which reflects peak gait impairment during the disease course. Patients with complex ataxia exhibited more severe disability (χ^2^ = 35.7, df = 8, *p* < 0.001). Cerebellar-dominant cases showed a wider distribution of severity, while the two non-cerebellar patients exhibited no gait impairment. Color coding: The red gradient in the leftmost column denotes increasing severity from “Normal” to “Wheelchair bounded”. Green shading within the table indicates the within-phenotype proportion for each severity category (darker shading represents a higher proportion). Background colors in the column headers are used only to distinguish phenotype groups.

Based on symptom constellations, patients were categorized into three major phenotypes (Figure 2B):

1. Pure cerebellar syndrome (*n* = 31)

This was the most prevalent form, comprising 14 Asian, 9 European, and 8 US patients. The phenotype was characterized by gait ataxia, nystagmus, and dysarthria, with variable additional neurological signs such as dysphagia, dysgeusia, sensory changes, oculomotor impairment. Onset age ranged broadly from 3 to 81 years (median 50, IQR 27–58.5). Most patients (54.8%) presented subacutely, while acute (16.1%) and chronic (6.5%) presentations were less frequent.

2. Cerebellar ataxia with encephalitic variant (*n* = 20)

This group included 13 European, 4 Asian, and 3 US patients. Patients displayed cerebellar features together with neuropsychiatric or cognitive impairment, often leading to more severe functional disability. Onset age ranged from 3 to 77 years (median 50, IQR 32–57.3). Subacute onset was again most frequent (50%), but acute (25%) and chronic (15%) courses were relatively more common than in the ataxia-dominant phenotype.

3. Non-cerebellar phenotype (*n* = 2)

This rare variant, reported exclusively in Europe, presented with encephalitic symptoms such as seizures and behavioral disturbances in the absence of cerebellar signs. Both cases occurred in middle-aged adults (49–50 years).

Severity of ataxia differed significantly across phenotypes (χ^2^ = 35.7, df = 8, *p* < 0.001). Patients in the complex ataxia group were predominantly wheelchair-dependent, whereas those in the ataxia-dominant group demonstrated a broader distribution of severity, and the two non-cerebellar patients had minimal ataxia. Regional analysis revealed a trend toward unsteady gait in patients reported from Asia and walker-dependence in patients reported from Europe, although significance was not reached.

### 3.3. Laboratory and Antibody Findings

CSF analysis revealed pleocytosis in 59.0% (23/39), elevated protein in 31.3% (5/16), and positive oligoclonal bands in 52.2% (12/23). Cytology, available in nine patients, showed lymphocytosis in three and mixed monocyte/heterokaryocyte responses in one, and was normal in five. CSF abnormalities did not correlate with phenotype or disease severity.

**Figure 3:**
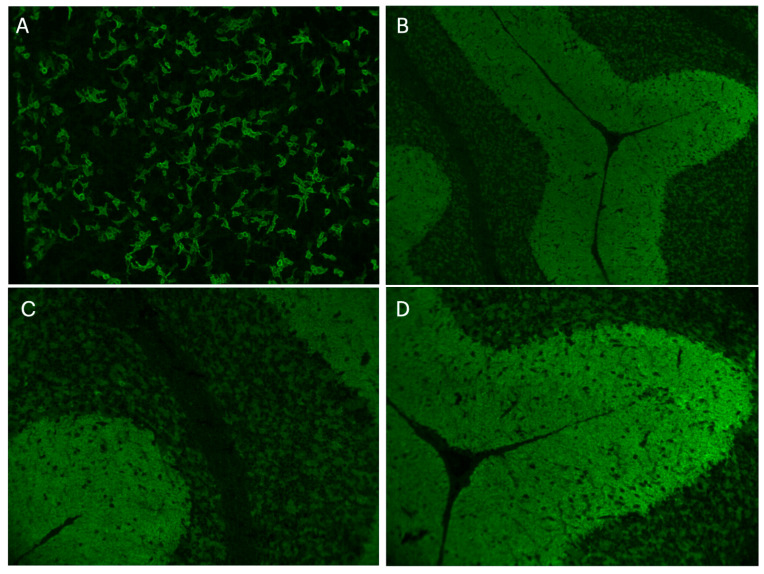
mGluR1 Antibody Staining in Transfected Cells and Tissue-Based Assay. (**A**) Cells transfected with the mGluR1 antibody exhibit positive staining, with an antibody titer of 1:320 (magnification: ×10). (**B**–**D**) The tissue-based assay shows positive staining in both the molecular layer and the Purkinje cell layer of the cerebellum. (**B**,**C**) are viewed at ×20 magnification, while (**D**) is at ×100 magnification.

Representative mGluR1 antibody staining results obtained by cell-based and tissue-based assays are shown in Figure 3. Antibody titers varied widely. Reported serum titers by tissue-based assay (TBA) ranged from 1:480 to 1:61,440, and by cell-based assay (CBA) from 1:20 to 1:12,800. CSF titers ranged from 1:64 to 1:512 (TBA) and 1:1 to 1:512 (CBA). In our newly tested cases (*n* = 11), positivity was confirmed by combined TBA+CBA, although precise titers were unavailable. Stratified analysis indicated significantly higher CBA-based serum titers in adults versus children (*p* = 0.025), but no differences were observed for CSF titers. Regional comparisons demonstrated lower TBA serum titers in patients reported from Asia (*p* = 0.029), whereas no differences were found by CBA.

### 3.4. MRI and EEG Findings

**Figure 4:**
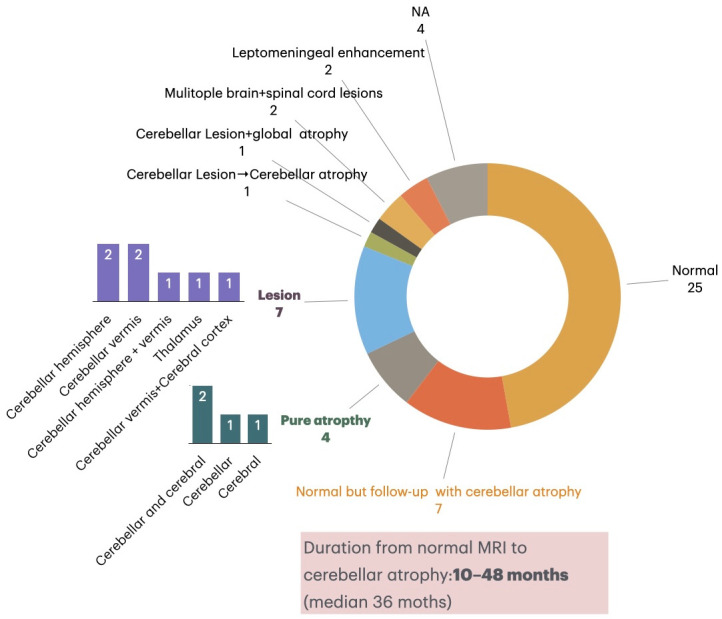
Neuroimaging Findings in mGluR1 Antibody Encephalitis Patients. Among 49 patients with available MRI data, 25 (49.0%) showed normal findings at onset, while 17 (34.7%) exhibited abnormalities. Seven patients demonstrated cerebellar lesions, most frequently involving the cerebellar hemispheres (*n* = 2), vermis (*n* = 2), or both (*n* = 1), and occasionally the thalamus or cerebral cortex. Four patients presented with pure cerebellar atrophy, and two showed leptomeningeal enhancement. Notably, seven patients with initially normal MRI later developed cerebellar atrophy during longitudinal follow-up, with a latency ranging from 10 to 48 months (median 36 months). These findings emphasize the progressive nature of structural changes and the importance of long-term imaging surveillance in anti-mGluR1 encephalitis.

Of 49 patients with MRI data, 17 (34.7%) demonstrated abnormalities (Figure 4). Pure cerebellar atrophy was present in 8.2% (4/49), focal lesions involving cerebellar hemispheres, vermis, thalamus, or cortex were seen in 14.3% (7/49), and two patients exhibited leptomeningeal enhancement. In addition, two patients developed multifocal brain and spinal cord lesions. Longitudinal follow-up revealed progression from initially normal imaging to cerebellar atrophy in 21.9% (7/32) patients over a median of 36 months, underscoring the dynamic course of structural changes. MRI abnormalities were not associated with antibody titers or assay type.

EEG results were available in 9/53 patients. Three showed normal background activity, while six presented with slowing-dominant abnormalities—diffuse theta–delta attenuation, frontal-predominant slow waves, focal hemispheric slowing, or sharp–slow complexes. No electrographic seizures or interictal epileptiform discharges were detected.

### 3.5. Treatment and Outcomes

**Figure 5:**
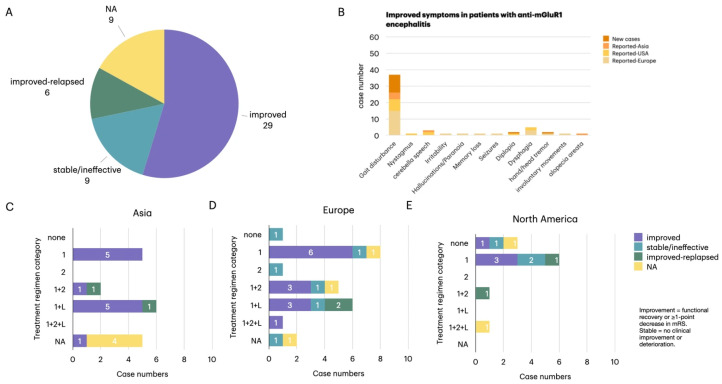
Treatment strategies and outcomes of patients with anti-mGluR1 encephalitis across regions. (**A**) Clinical outcomes were available for 44 patients: 29 (65.9%) improved, 9 (20.5%) remained stable or unaffected, and 6 (13.6%) experienced relapse after initial improvement; 9 lacked follow-up data. (**B**) Symptom-specific improvements were most frequently observed in gait disturbance, nystagmus, and dysarthria. (**C**–**E**) Regional comparison of immunotherapy regimens in Asia, Europe, and North America. Treatment categories are defined as: 1 = first-line (high-dose corticosteroids, IVIG, or plasma exchange); 2 = second-line (rituximab or cyclophosphamide); L = long-term maintenance (mycophenolate mofetil, azathioprine, or tacrolimus). Combination regimens are denoted as 1 + 2, 1 + L, or 1 + 2 + L. Asian patients more frequently received first-line therapy with long-term maintenance, European patients tended toward combination regimens, and North American patients primarily relied on first-line therapy alone. Overall, regional differences in therapeutic strategy were statistically significant (*p* = 0.041).

Therapeutic data were available for 44 patients. Treatment strategies and outcomes are summarized in Figure 5. First-line immunotherapy (glucocorticoids, IVIG, or PLEX) was administered in 21 cases, while 22 required escalation to second-line or long-term immunosuppressants. Follow-up duration varied widely across cases, ranging from 10 weeks to 14 years, with the majority falling within 6–24 months. Overall, 65.9% (29/44) achieved clinical improvement, 13.6% (6/44) relapsed, and 20.5% (9/44) showed stable or no response.

Treatment regimens varied significantly by region (*p* = 0.041). Patients reported from Asia most often received first-line therapy alone or in combination with long-term immunosuppressants. patients reported from Europe demonstrated the most heterogeneous approaches, including triple therapy and combinations of first- and second-line agents. In contrast, patients reported from North America were largely treated with first-line regimens, with a notable proportion receiving no therapy. Despite these differences, overall response rates were comparable across regions (*p* = 0.48).

Patients with comorbid tumors demonstrated variable responses. Of seven receiving immunotherapy, five improved and two did not respond. Outcomes in patients treated for underlying malignancies (chemotherapy or surgery) were mixed, with some improving and others deteriorating. Tumor-associated cases tended to exhibit milder baseline ataxia, though overall prognosis did not clearly differ from non-tumor cases.

## 4. Discussion

Our study presents the largest integrated cohort of anti-mGluR1 encephalitis to date, combining 11 newly identified Chinese cases with 42 published reports worldwide [4,5,6,7,8,9,10,11,12,13,14,15,16,17,18,19,20,21,22,23]. By analyzing these 53 patients, we update the current understanding of the clinical spectrum, diagnostic features, therapeutic response, and outcomes of this rare autoimmune encephalitis. Importantly, the strength of this work lies not only in sample size but also in analytical depth. Beyond enlarging the cohort, our study advances prior literature by incorporating structured phenotype clustering with longitudinal MRI tracking and by providing cross-publication regional comparisons of treatment selection and prognosis.

### 4.1. Clinical and Pathophysiologic Spectrum

In our integrated cohort, the overwhelming majority presented with acute to subacute cerebellar ataxia, consolidating the view that cerebellar dysfunction is the clinical hallmark of anti-mGluR1 encephalitis [4,6,10]. Similarly to Chen et al. [28], we confirmed ataxia as the dominant presentation, but our data further extend the spectrum by identifying dysgeusia and transient encephalitic manifestations, features rarely emphasized in prior reviews [7,15]. Supplementary Figures S2 and S3 further illustrate demographic and phenotypic patterns across the pooled cohort. Most published cases showed an adult predominance and pure ataxia at onset, while severity and treatment patterns varied across publication regions. Mechanistically, these atypical symptoms are consistent with the distribution of mGluR1 across neural networks: high receptor density in the cerebellar cortex explains gait and coordination deficits, while disruption of cerebello–cerebral loops may produce cognitive–affective disturbances (CCAS) [29,30]. Expression in hippocampus and thalamus provides a substrate for psychiatric symptoms and seizures [5,31], and in basal ganglia and posterior tongue may underlie dysgeusia [31]. Compared with Chen et al., who highlighted classical cerebellar and degenerative phenotypes, our findings suggest a broader pathophysiologic involvement and observed regional patterns are more likely attributable to reporting or clinical practice differences than to inherent biological modifiers. Psychiatric features—more often reported in European cohorts of autoimmune encephalitis [5,10,31,32]—also emerged in some mGluR1 cases. Pediatric cases remained rare; while no clear adult–pediatric differences were evident, children in our series more often exhibited acute movement disorder–like presentations, echoing earlier observations [11,12]. These findings suggest that anti-mGluR1 antibodies may exert both synaptic dysfunction and delayed neurodegenerative effects, consistent with emerging models of antibody-mediated cerebellar autoimmunity [10,27].

In our cohort, the median time from symptom onset to confirmed diagnosis varied markedly, and in several patients, the diagnostic process extended close to one year. This may relate to the initially mild or non-specific symptoms, limited early recognition of anti-mGluR1 encephalitis, and delayed antibody testing. These findings highlight the need for increased clinical awareness and earlier autoimmune screening in patients with unexplained ataxia or dysarthria.

### 4.2. Neuroimaging and Laboratory Findings

Neuroimaging of the patients with anti-mGluR1 encephalitis indicated a progressive course. Many patients had normal baseline MRI yet developed cerebellar atrophy on longitudinal scans, possibly consistent with delayed structural injury such as Purkinje cell loss, although causality cannot be established from current data. We did not observe a robust clinicoradiologic correlation, in contrast to some prior series [6,9], and underscoring the importance of timely and sustained immunotherapy even when initial imaging appears unremarkable. For our PUMCH cohort, follow-up MRI has not yet been performed at the current data cutoff; therefore, progression and atrophy evolution could not be evaluated. Only baseline MRI findings are presented, and longitudinal imaging outcomes will be captured in ongoing follow-up. And the EEG changes were mostly non-epileptiform, suggesting cortical dysfunction rather than seizure-driven pathology, consistent with the low seizure frequency observed in anti-mGluR1 encephalitis.

Findings of CSF analyses were variable. Mild pleocytosis or protein elevation was observed in several patients, yet no consistent pattern emerged, aligning with earlier studies [9,14]. A key point is that the timing of lumbar puncture was not standardized in the published literature; in many reports, it was unclear whether CSF was obtained at symptom onset or at the time of confirmed diagnosis. These variations may partially explain the inconsistencies in pleocytosis rates and warrant cautious interpretation when comparing data across studies.

In our cohort, all PUMCH patients underwent lumbar puncture during the diagnostic work-up, providing greater internal consistency within this subgroup. Importantly, all patients tested positive for anti-mGluR1 antibodies, typically confirmed by cell-based assay (CBA), reaffirming antibody testing as the diagnostic gold standard [4,16].

### 4.3. Treatment Response and Outcomes

First-line immunotherapy was effective in most patients, consistent with prior reports [6,17] and the review by Chen et al. [28], which similarly emphasized favorable responses to early treatment. However, in our cohort more than one-third required escalation to long-term immunosuppressants such as mycophenolate mofetil or rituximab, reflecting a treatment-refractory subgroup also noted in European and North American cohorts [10,19]. Notably, although all PUMCH cases achieved clinical improvement, residual deficits—mainly gait imbalance—remained common, underscoring a persistent functional burden despite apparent immunological remission. This observation is consistent with other series reporting incomplete recovery despite immunological remission [8,20]. No deaths were observed, reinforcing that prognosis is favorable when diagnosis and treatment are timely. Stratified analysis of antibody titers showed no correlation with treatment outcomes in CBA testing. In TBA, however, higher CSF titers were associated with poorer outcomes (ρ = −0.71, *p* = 0.047), although the small sample size and methodological variability limit interpretation.

### 4.4. Tumor Association and Regional Differences

Malignancy was present in 9 of 53 patients (17.0%), most commonly lymphoma (*n* = 6), with single cases of seminoma, leukemia, sarcoma, and prostate adenocarcinoma. Although not present in the majority, this proportion is clinically meaningful for a rare antibody, and routine tumor surveillance should be considered. Given this background, we recommend baseline malignancy screening for all newly diagnosed cases, including CT or PET-CT when clinically appropriate, and periodic re-evaluation during follow-up, especially in patients with progressive ataxia or incomplete therapeutic response. Some tumor-associated cases improved with immunotherapy and/or oncologic treatment, whereas others deteriorated. Baseline ataxia severity tended to be milder in tumor-associated disease, but outcomes were heterogeneous and numbers small; definitive inferences about the effect of immunotherapy in paraneoplastic contexts require larger cohorts.

Therapeutic strategies differed by region (*p* = 0.041): Asian cohorts favored long-term maintenance therapy, European cohorts used more complex combinations, and North American cohorts relied primarily on first-line regimens. Such differences likely reflect healthcare system structure, drug accessibility, and diagnostic timing rather than biological variability.

Notably, “geographic differences” in this study reflect the regions where cases were published, not necessarily patients’ origin. Therefore, differences in onset age, phenotype or treatment likely reflect reporting patterns, diagnostic accessibility, or healthcare systems, rather than confirmed biological variation

## 5. Limitations

This study integrates a single-center case series with published cases, which introduces reporting and selection biases, regional heterogeneity in diagnostic depth, and variable follow-up duration. Sample sizes for certain subgroup analyses (e.g., titer–outcome correlations, pediatric cases) were small, and some laboratory denominators differed across measures. Regional comparisons should be interpreted cautiously because the dataset is grouped by publication region rather than true geographic origin of patients. Multiple comparisons were conducted without adjustment for multiplicity, which increases the risk of type I error. Reported statistical differences should be interpreted cautiously, and findings are exploratory pending validation in larger cohorts. Future prospective, multicenter registries with standardized antibody assays, imaging protocols, and outcome measures are needed to validate these observations.

## 6. Conclusions

This study expands the clinical spectrum of anti-mGluR1 encephalitis, confirming cerebellar ataxia as the hallmark while identifying additional features such as dysgeusia and encephalitic symptoms, consistent with and extending prior reports [4,6,10,28]. Moreover, we provided a more refined stratification of clinical phenotypes and quantified differences in severity and disability across subgroups. Anti-mGluR1 antibodies appear pathogenic rather than “low-risk,” with paraneoplastic associations relatively uncommon. Early immunotherapy was generally effective, with regional differences observed—our Chinese cohort showed younger onset and greater reliance on long-term immunosuppression compared with later-onset Western cases [28]. Looking forward, standardized antibody testing, multicenter registries, and longitudinal biomarker studies will be critical to define treatment windows, evaluate relapse predictors, and establish evidence-based immunotherapy algorithms. Early recognition and sustained immunosuppression remain essential to prevent irreversible cerebellar damage.

## Figures and Tables

**Table 1:** Clinical characteristics, ancillary findings, treatment, and outcomes of 11 patients with anti-mGluR1 encephalitis from PUMCH.

Case	Age/Sex	Duration to Diagnosis	Clinical Manifestation	Ataxia Severity (Baseline)	CSF (WBC/Protein/OCB)	MRI Findings	Antibody (Serum/CSF, Method)	Immunotherapy (1st-Line)	2nd-Line/Maintenance	Treatment Response	Ataxia Severity (Follow-Up)
1	41/M	1 yr	Ataxia	Wheelchair-dependent	Pleocytosis (9), NA, NA	NA	320/320, CBA	MP(S) + IVIG	MMF	Improvement	Unsteady gait
2	50/M	2 mo	Ataxia	Wheelchair-dependent	Pleocytosis (50–190), NA, NA	NA	100/NA, CBA	MP + IVIG	MMF	Improvement	Unsteady gait
3	33/M	3 mo	Fear sensations, ataxia	Unsteady gait	Pleocytosis (15), NA, NA	NA	100/100, CBA	MP(S)	—	Improvement	NA
4	46/M	10 d	Ataxia, dysgeusia	Walker required	Pleocytosis (16), OCB+	WM T2 hyperintensity	10/100, CBA	IVIG (ineffective) → MP(S)	MMF	Improvement	Normal
5	46/F	1 yr	Ataxia	Walker required	Pleocytosis (9)	NA	320/320, CBA	MP(S) + IVIG	MMF	Improvement	Unsteady gait
6	57/M	4 mo	Ataxia	Unsteady gait	WBC neg, OCB+	NA	320/100, CBA	MP(S) + IVIG	Rituximab	Improvement	Unsteady gait
7	30/M	1 mo	Ataxia	Unsteady gait	WBC neg, OCB+	NA	320/100, CBA	MP(S) + IVIG	—	Improvement	Unsteady gait
8	23/F	2 mo	Ataxia	Unsteady gait	NA	R cerebellar hyperintensity	Positive (serum/CSF), CBA	MP(S) + IVIG	—	Improvement	Unsteady gait
9	57/F	1 mo	Ataxia	Walker required	Pleocytosis (13), protein 293	NA	320/320, CBA	MP (ineffective) → IVIG	—	Improvement	Unsteady gait
10	60/M	4 mo	Ataxia	Walker required	Protein 530, OCB+	NA	320/100, CBA	IVIG + MP	—	Improvement	NA
11	61/F	4 mo	Confusion, dysgeusia	Wheelchair-dependent	Pleocytosis (525→175), protein elevated protein, OCB+	Cerebellar T2/DWI hyperintensity	320/320, CBA	MP + IVIG	Rituximab	Stable → Improvement	NA

## Data Availability

Data supporting the findings of this study are available from the corresponding author upon reasonable request.

## Supplementary Materials

The following supporting information can be downloaded at: https://www.mdpi.com/article/10.3390/diagnostics16020321/s1, Figure S1: Flow diagram of the literature selection process for the pooled analysis. Figure S2: Geographic and age distribution of anti-mGluR1 encephalitis cases. Figure S3: Severity and onset pattern of anti-mGluR1 encephalitis across regions and age groups. Table S1: Individual-level clinical features, treatment, and outcomes of anti-mGluR1 encephalitis patients.

## Abbreviations

MP(S) = methylprednisolone pulse; MMF = mycophenolate mofetil; RTX = rituximab; CTX = cyclophosphamide; IVIG = intravenous immunoglobulin; OCB = oligoclonal bands; NA = not available; mRS = modified Rankin Scale; MRI = magnetic resonance imaging; CSF = cerebrospinal fluid.
