# Supplementary material for: Clinical Phenotypes and Prognosis of Anti-mGluR1 Encephalitis: A Single-Center Case Series and Comprehensive Literature Review
Source: Diagnostics (Basel). 2026 Jan 19;16(2):321. doi: 10.3390/diagnostics16020321 (PMC12839809; doi:10.3390/diagnostics16020321)
Supplement: Supplementary file 1 [file diagnostics-16-00321-s001.zip › diagnostics-3969078-supplementary.pdf]

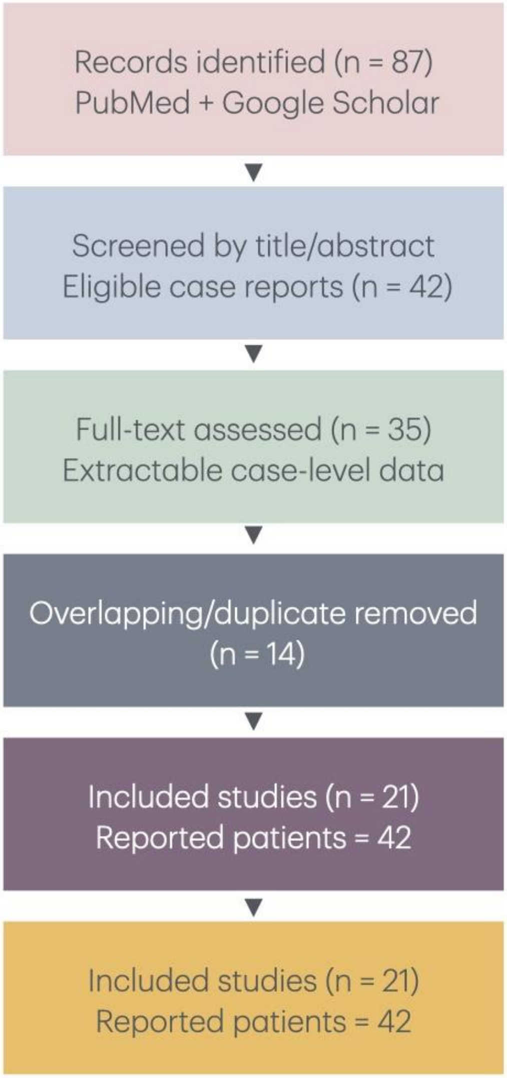

Figure S1. Flow diagram of the literature selection process for the pooled analysis. A total of 87 records were identified, 21 studies containing 42 unique patients were included, together with 11 PUMCH cases, resulting in a final pooled dataset of 53 patients.

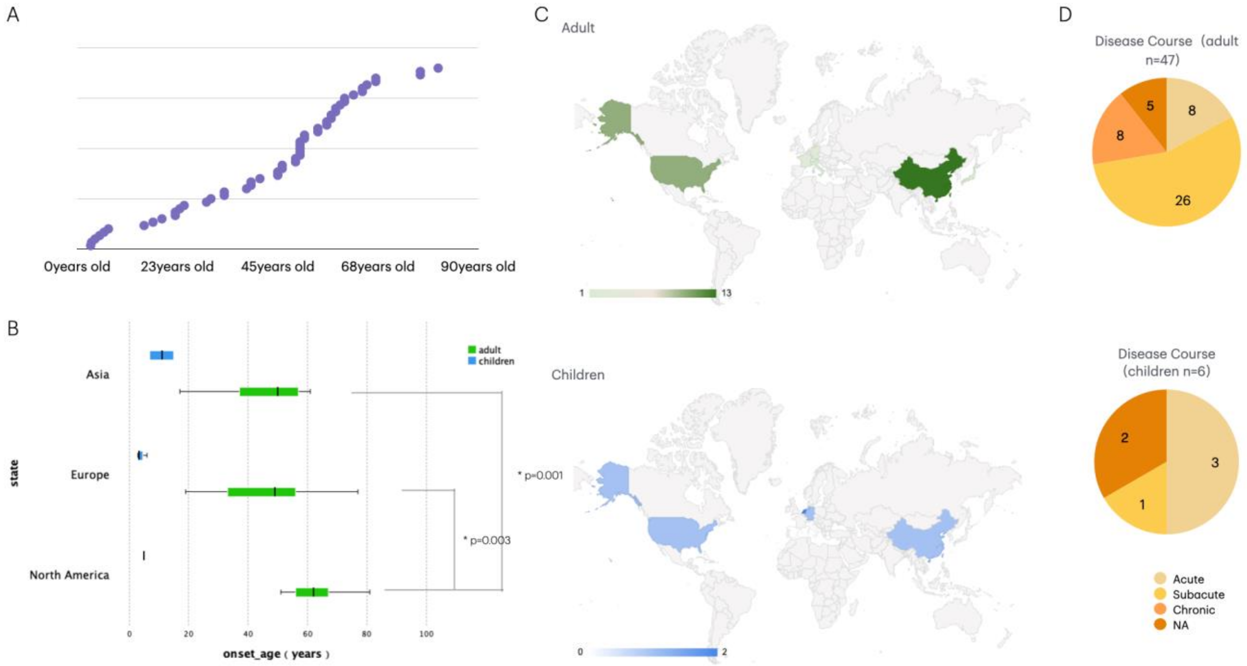

Figure S2. Geographic and age distribution of anti-mGluR1 encephalitis cases. A: Age distribution of the 53 included patients, ranging from 3 to 81 years, demonstrates a bimodal pattern with a predominant adult peak. B: Comparison of onset age stratified by region (Asia, Europe, North America) and age group revealed that adult patients in North America had a significantly later disease onset compared with their Asian ($p = 0.001$) and European ($p = 0.003$) counterparts, whereas no significant regional difference was observed among pediatric cases, likely due to the limited sample size. C: Global distribution maps of adult ($n = 47$, shown in green) and pediatric ($n = 6$, shown in blue) cases. D: Disease course in adults was acute in 8 (17%), subacute in 26 (55%), and chronic in 8 (17%) patients, while among children, 3 (50%) had subacute onset, 2 (33%) had chronic courses, and 1 (17%) presented acutely.

A

Severity	Asia	USA	Europe
Normal	0/14	11.1%(1/9)	15%(3/20)
Unsteady gait	50%(7/14)	22.2%(2/9)	10%(2/20)
Walker required	35.7%(5/14)	55.5%(5/9)	60%(12/20)
Wheelchair bounded	14.3%(2/14)	11.1%(1/9)	15%(3/20)

B

Severity	Children	Adult
Normal	0/3	10%(4/40)
Unsteady gait	33.3%(1/3)	25%(10/40)
Walker required	66.7%(2/3)	50%(20/40)
Wheelchair bounded	0/3	15%(6/40)

C

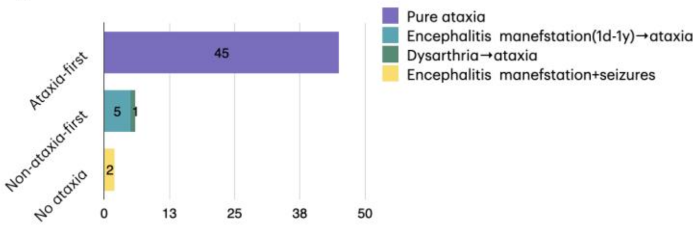

Figure S3. Severity and onset pattern of anti-mGluR1 encephalitis across regions and age groups. A: Severity of ataxia at peak disease stratified by region. Most European patients exhibited moderate to severe ataxia, with 60% requiring walking aids and 15% wheelchair-bound, compared to milder presentations in Asian (50% unsteady gait) and American (55.5% walker required) cohorts. B: Severity comparison between adults and children. Adults tended to experience more severe ataxia overall (50% walker required, 15% wheelchair-bound), whereas two-thirds of pediatric patients required walking aids but none were wheelchair-dependent. C: Distribution of onset patterns. Among 52 evaluable cases, 45 (86.5%) presented with pure ataxia as the initial symptom, 5 (9.6%) initially exhibited encephalitic features before developing ataxia, 1 (1.9%) progressed from dysarthria to ataxia, and 2 (3.8%) had encephalitic-onset seizures without subsequent ataxia.
